# A novel G-quadruplex motif in the Human *MET* promoter region

**DOI:** 10.1042/BSR20171128

**Published:** 2017-11-29

**Authors:** Jing Yan, Deming Zhao, Liping Dong, Shuang Pan, Fengjin Hao, Yifu Guan

**Affiliations:** 1 Department of Biochemistry and Molecular Biology, China Medical University, Shenyang, Liaoning 110122, China; 2 Department of Gastrointestinal Surgery, Dalian Municipal Central Hospital, Dalian, Liaoning 116033, China; 3 Library of China Medical University, Shenyang, Liaoning 110122, China; 4 Department of Physiology, Jinzhou Medical University, Liaoning 121001, China

**Keywords:** G-quadruplex, MET promoter, TMPyP4

## Abstract

It is known that the guanine-rich strands in proto-oncogene promoters can fold into G-quadruplex structures to regulate gene expression. An intramolecular parallel G-quadruplex has been identified in *MET* promoter. It acts as a repressor in regulating *MET* expression. However, the full guanine-rich region in *MET* promoter forms a hybrid parallel/antiparallel G-quadruplex structure under physiological conditions, which means there are some antiparallel and hybrid parallel/antiparallel G-quadruplex structures in this region. In the present study, our data indicate that g3-5 truncation adopts an intramolecular hybrid parallel/antiparallel G-quadruplex under physiological conditions *in vitro*. The g3-5 G-quadruplex structure significantly stops polymerization by Klenow fragment in K^+^ buffer. Furthermore, the results of circular dichroism (CD) spectra and polymerase stop assay directly demonstrate that the G-quadruplex structure in g3-5 fragment can be stabilized by the G-quadruplex ligand TMPyP4 (5,10,15,20-tetra-(N-methyl-4-pyridyl) porphine). But the dual luciferase assay indicates TMPyP4 has no effect on the formation of g3-5 G-quadruplex in HepG2 cells. The findings in the present study will enrich our understanding of the G-quadruplex formation in proto-oncogene promoters and the mechanisms of gene expression regulation.

## Introduction

*MET* proto-oncogene encodes the hepatocyte growth factor receptor (HGFR or MET), which can be activated by its ligand hepatocyte growth factor (HGF). METs are involved in several diverse physiological events through Ras/MAPK, PI3K/Akt, c-Srcand STAT3/5 signaling pathways [1]. However, abnormal activation of MET can result in cell proliferation, antiapoptosis, angiogenesis, altered cytoskeletal function, metastasis and poor prognosis [2–5]. In many types of human malignancies, including cancers of kidney, liver, stomach, breast and brain, METs are abnormally activated and can promote tumor growth, angiogenesis and cancer metastasis [4]. Hence, *MET* becomes an attractive target for cancer therapy [6,7].

G-quadruplex structure can be formed in single-stranded guanine-rich nucleic acid fragments, which is a kind of noncanonical secondary structure. It is a four-stranded nucleic acid that contains stacked G-quartets of four guanines connected by Hoogsteen hydrogen bonds [8,9]. In the human genome, these G-quadruplex structures can play different roles. Recently, several studies have revealed that G-quadruplex structures in proto-oncogene promoters are capable of regulating gene transcription and can be used as candidates for cancer therapy [10–17]. The G-quadruplex ligand TMPyP4 binds and stabilizes G-quadruplexes in human promoter sequences, resulting in inhibition of gene transcription [11,12,17–19]. Currently, TMPyP4 and its analogs are under study as potential anticancer agents.

**Figure 1:**
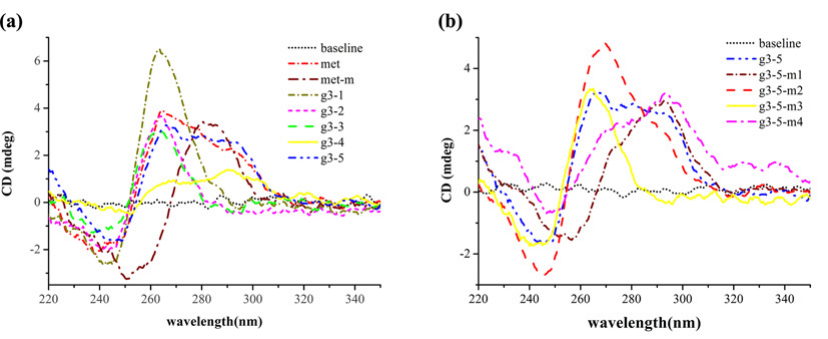
CD spectra of the truncations of met and mutants of g3-5 Each spectrum is collected at a final concentration of 5 μM oligonucleotide in a sodium cacodylate buffer (20 mM, pH 7.4) containing 140 mM KCl. (**a**) CD spectra of the truncations in met sequence. (**b**) CD spectra of g3-5 and its mutants.

Our previous research has discovered that Pu23WT fragment in *MET* promoter (-48 to -26 upstream of transcription start site) can adopt an intramolecular parallel G-quadruplex structure, which functions as a transcriptional repressor of *MET* [20]. Moreover, the cationic porphyrin TMPyP4 can stabilize the Pu23WT G-quadruplex. It has been shown that Pu23WT G-quadruplex could be a candidate target for anticancer treatment. However, CD spectrum in the present study showed that the entire G-rich strand in *MET* proximal promoter (met, -91 to -26 upstream of transcription start site) folded into a hybrid parallel/antiparallel G-quadruplex structure under physiological conditions (Table 1 and Figure 1), which means there are some potential antiparallel or hybrid parallel/antiparallel G-quadruplex structures in this region. These relationships led us to truncate the met strand into several short fragments (Table 1) to investigate the G-quadruplex structures and their biological effects on *MET* transcription.

**Table 1:** Summary of ODNs used in the present study

Name	Sequence
met	CAGGGCAGCGCGCGTGTGGGAAGGGGCGGAGGGAGTGCGGCCGGCGGGCGGGCGGGGCGCTGGGCT
met-m	CAG**T**GCAGCGCGCGTGTG**T**GAAG**TT**GCGGAG**T**GAGTGCGGCCGGCG**T**GCG**T**GCG**TT**GCGCTG**T**GCT
g3-1/Pu23WT	GCGGGCGGGCGGGGCGCTGGGCT
g3-2	GAGGGAGTGCGGCCGGCGGGCGGGCGGGGCG
g3-3	AGGGGCGGAGGGAGTGCGGCCGGCGGGCGGGCG
g3-4	GTGGGAAGGGGCGGAGGGAGTGCGGCCGGCGGGCG
g3-5	CAGGGCAGCGCGCGTGTGGGAAGGGGCGGAGGGAG
g3-5-m1	CAGGGCAGCGCGCGTGTGGGAA**T**GGGCGGAGGGAG
g3-5-m2	CAGGGCAGCGCGCGTGTGGGAAGGG**T**CGGAGGGAG
g3-5-m3	CAG**TT**CAGCGCGCGTGTGGGAAGGGGCGGAGGGAG
g3-5-m4	CAGGGCAGCGCGCGTGTGGGAAGGGGCGGA**TT**GAG

G-islands were underlined. The guanine (G) was replaced in G-islands by the thymine (T) in bold.

## Methods

### Preparation of oligonucleotides

The DNA sequence of human *MET* promoter was acquired in GenBank database [GenBank GI: 224589819, NC_000007.13]. The truncated oligonucleotides and their mutants were synthesized by Sangon Biotech Co., Ltd. (Shanghai, China). The potentiality of G-quadruplex formation was evaluated by the online software Quadparser (http://www.quadruplex.org/?view=quadparser). The oligonucleotides were prepared at 5 μM in 20 mM sodium cacodylate with a 140 mM KCl buffer (pH 7.4) unless specified otherwise.

### Circular dichroism spectroscopy and ultraviolet absorption spectroscopy

The CD spectra and the ultraviolet (UV) absorbance spectra at 295 nm were acquired according to the procedures described in our previous study [20]. A slow melting–annealing cycle was performed prior to each CD and UV measurements.

The CD spectra were recorded over a wavelength range of 220–350 nm at room temperature for at least three independent scans. They were baseline-corrected by subtracting the signal contributions of the buffer. The 5 μM oligonucleotide samples were prepared with different concentrations of KCl, NaCl, MgCl_2_, CaCl_2_, and TMPyP4. When recording the TMPyP4-dependent CD spectra, the samples were prepared with 10 mM KCl.

The UV absorbance spectra at 295 nm were used to analyze the thermal stability of samples. The spectra were recorded between 20 and 80°C with 1°C increment during heating and cooling processes. A slow heating/cooling rate of 0.3°C/min was applied to ensure the thermal transition under an equilibrium condition. The melting and annealing curves were averaged with three independent measurements. The data were normalized by the formula: *F*_F_(*θ*) = (*Y* − *Y*_min_)/(*Y*_max_− *Y*_min_), where *θ* is the fraction of folded G-quadruplex at a given temperature, *Y* is the signal at 295 nm. The *T*_m_ or *T*_a_ values were calculated according to the normalized profiles (fraction folded profiles) at *θ* = 0.5 [21].

### Non-denatured electrophoresis

g3-5 and its mutants were denatured at 95°C for 10 min in the 140 mM KCl, then cooled down slowly to room temperature and left overnight. All the samples were analyzed in 20% non-denatured polyacrylamide gels at 4°C, which were stained with Stains-all (Sigma-Aldrich, U.S.A.) in darkness. The standards were the 35 nt single-stranded oligonucleotide and the 20 bp double-stranded oligonucleotide. The images were recorded using a Personal Scanner (FangZheng, China).

### Polymerase stop assay

The g3-5 strand was linked with either a long (AGCCTAGCTGCAAGTATGTTCACG) or short (ATGTTCACG) tail sequence at the 3′-end to formulate templates for LTg3-5 and Tg3-5 (Table 2). The primer 5′-d(ATCCAGAAGTACGTGAACAT)-3′ contained a complementary sequence to LTg3-5 and Tg3-5 tails [22]. LTg3-5 or Tg3-5 was denatured with primer then annealed to form asymmetric duplex. The two-way-extension of the asymmetric duplex by Klenow fragments was kept for 30 min at room temperature. We then employed the same procedure shown in our previous work [20]. The two-way-extension can be stopped by the G-quadruplex in the template.

**Table 2:** Templates and primer used in DNA polymerase stop assay

Name	Sequence
g3-5	CAGGGCAGCGCGCGTGTGGGAAGGGGCGGAGGGAG
LTg3-5	CAGGGCAGCGCGCGTGTGGGAAGGGGCGGAGGGAG***AGCCTAGCTGCAAG******T****ATGTTCACG***
Tg3-5	CAGGGCAGCGCGCGTGTGGGAAGGGGCGGAGGGAG***ATGTTCACG***
primer	ATCCAGAAGTA**CGTGAACAT**

The additional tail sequences of g3-5 were shown as italic.

The complementary sequences between template and primer were underlined.

### Dual luciferase assay

Recombinant DNA 0.7met-pGL4.10 was the firefly luciferase reporter driven by *MET* promoter, which contained the full G-rich region within *MET* promoter [20]. Site-directed mutations in met strand (G to T, Table 3) were performed using the QuikChange^®^ Lightning Site-Directed Mutagenesis Kit (Stratagene, U.S.A.). To assess the effect of TMPyP4 on *MET* promoter activity, HepG2 cells were separately transfected with 0.7met-pGL4.10 constructor, the mutant g3-1-mut, and the mutant g3-5-mut. The post-transfected HepG2 cells were treated with 50 μM TMPyP4 for 24 h. The cells were protected from light during the period of TMPyP4’s treatment. The activities of firefly luciferase and renilla luciferase were assayed by the Dual Luciferase Reporter Assay System E1910 (Promega, U.S.A.) on Infinite M200 Multi-plate Reader (Tecan, Switzerland). Three independent experiments were done in triplicate.

**Table 3:** Site-directed mutations of g3-1 and g3-5

Name	Sequence
g3-1/Pu23WT	GCGGGCGGGCGGGGCGCTGGGCT
g3-1-mut	GCGGGCGGGCG**T**GGCGCTG**T**GCT
g3-5	CAGGGCAGCGCGCGTGTGGGAAGGGGCGGAGGGAG
g3-5-mut	CAG**T**GCAGCGCGCGTGTGGGAAG**T**GGCGGAG**T**GAG

The guanine (G) was replaced in G-islands by the thymine (T) in bold.

### Statistical analysis

The data were presented as the mean ± SD in at least three separate experiments. Statistical analysis was performed using one-way ANOVA by GraphPad Prism 5.0 software. The statistically significant difference was defined as *P*<0.05.

## Results and discussion

### g3-5 forms an intramolecular hybrid antiparallel/parallel G-quadruplex structure in K^+^ solution

The met strand in *MET* approximal promoter and its truncated fragments were listed in Table 1. Each truncation was composed of at least four consecutive G-islands. A serial of mutants (G to T) of g3-5 were used to decipher which Gs were potentially involved in the formation of G-quadruplex structure.

We recorded CD spectra of the met strand and its truncated fragments in 140 mM K^+^ buffer (pH 7.4) (Figure 1a). The CD spectrum of met exhibited a positive peak at 264 nm, a negative peak at 240 nm, and a shoulder peak approximately 290 nm, indicating the formation of a hybrid parallel/antiparallel G-quadruplex structures. The spectrum of met-m (mutant of met) showed a positive peak at 280 nm and a negative peak at 250 nm. There was no characteristic CD spectrum in met-m. Truncation g3-1/Pu23WT, g3-2, and g3-3 exhibited a positive peak approximately 260 nm and a negative peak approximately 240 nm. In terms of the CD spectra, they all adopted parallel G-quadruplex structures. The truncation of g3-4 did not show any characteristic of G-quadruplex spectrums. However, the CD spectrum of g3-5 was uniform with that of met, exhibiting a positive peak at 264 nm, a negative peak at 240 nm, and a shoulder peak approximately 290 nm. Both of them formed a hybrid parallel/antiparallel G-quadruplex structure. Due to the hybrid parallel/antiparallel G-quadruplex in the full G-rich strand of met, there had to contain antiparallel or hybrid parallel/antiparallel G-quadruplex structures. According to CD spectra, only g3-5 was able to adopt a hybrid parallel/antiparallel G-quadruplex structure. Moreover, g3-5 truncation got high score when assessing the ability to fold into G-quadruplex via the online software Quadparser. In addition, UV absorbance spectra of g3-2, g3-3, and g3-4 showed no G-quadruplex structures in these strands (Supplementary Figure S1). Hence, g3-5 and g3-1/Pu23WT made contributions to the formation of the hybrid parallel/antiparallel G-quadruplex in *MET* promoter.

The g3-5 contained five G-islands, four of them contain three or four guanines. Only one G-island was composed of GG repeat (Table 1). Consequently, there would be several ways to form G-quadruplex structure using different G-islands. In order to decipher the number of stacked G-tetrads and the guanines involved in g3-5 G-quadruplex structure, we compared the CD profiles of g3-5 and its mutants (Figure 1b). The CD spectra were collected in the same conditions. The mutant g3-5-m2 exhibited a very similar CD spectrum with that of g3-5, suggesting a hybrid parallel/antiparallel G-quadruplex structure in K^+^ solution. The CD spectrum of g3-5-m3 showed a positive peak approximately 260 nm and a negative peak approximately 240 nm, which indicated a parallel G-quadruplex structure. Other mutants could not fold into any G-quadruplex structure according to their CD spectra. Therefore, the Gs in the g3-5-m2 mutant formed the hybrid parallel/antiparallel G-quadruplex structure of g3-5 in K^+^ buffer.

**Figure 2:**
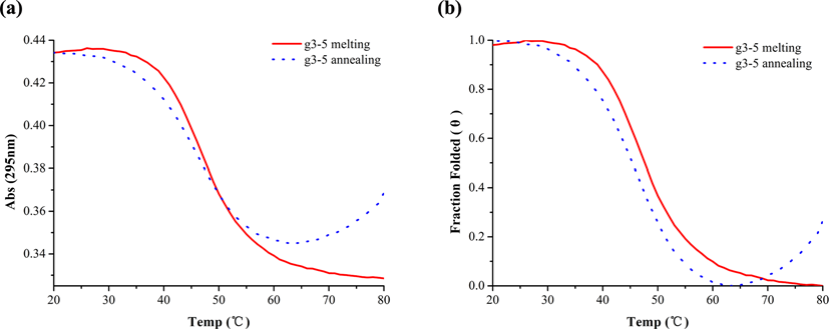
The melting and annealing curves of g3-5 at 295 nm (**a**) The melting and annealing curves of g3-5 at 295 nm. (**b**) The normalized melting and annealing curves of g3-5 at 295 nm.

In the K^+^ buffer, the absorbance spectra at 295 nm of g3-5 showed a hypochromic effect except a small evaporation artifact between the melting and annealing curves (Figure 2a), which suggested the process was indeed in thermodynamic equilibrium. This reversible phenomenon supported the formation of an intramolecular G-quadruplex. *T*_m_ and *T*_a_ values of g3-5 in 140 mM KCl were determined by the folded fraction profiles at *θ* = 0.5 (Figure 2b), which were 47.46 ± 0.27 and 45.64 ± 0.31°C. The *T*_m_ value of g3-5 was much lower than that of g3-1/Pu23WT (81.07 ± 0.35°C) in 140 mM KCl [20].

**Figure 3:**
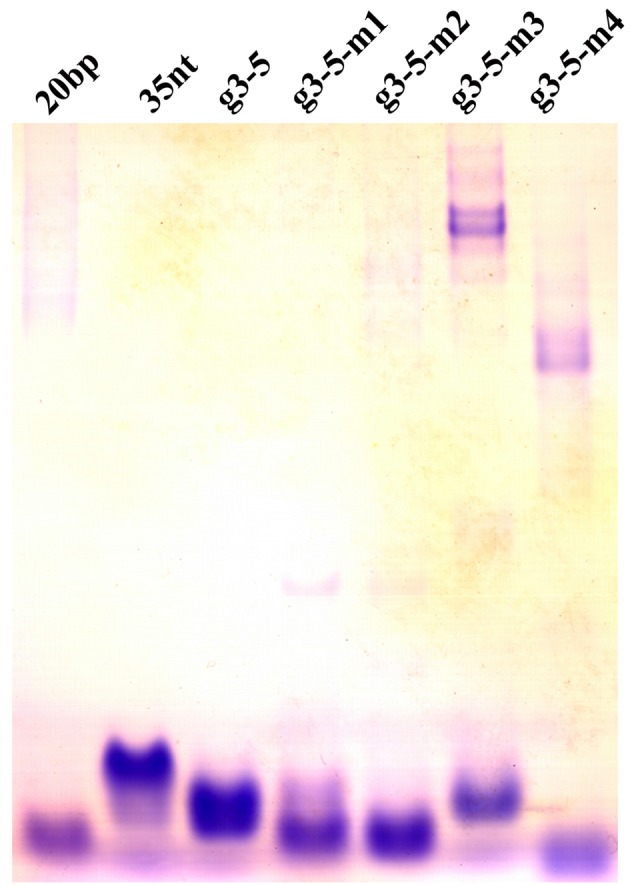
Non-denatured PAGE data of g3-5 and its mutants Non-denatured PAGE of g3-5 and its mutants in the presence of 140 mM KCl. The lanes 1 and 2 show the double-stranded and single-stranded molecular-weight size markers.

The mobilities of g3-5 and its mutants were printed on the non-denatured polyacrylamide gel containing 140 mM KCl at 4°C. The g3-5 strand migrated faster than the 35 nt control strand (lane 3, Figure 3), indicating that g3-5 folded into a compact intramolecular structure, and g3-5-m2 ran faster than g3-5, which also appeared as a single species (lanes 5, Figure 3).

In conclusion, the g3-5 strand could fold into an intramolecular hybrid parallel/antiparallel G-quadruplex structure by using the Gs in the g3-5-m2 mutant in K^+^ buffer. It was assumed that g3-5 and g3-1/Pu23WT G-quadruplexes coexisted in met strand, resulting in a hybrid parallel/antiparallel G-quadruplex, even though the loops in this G-quadruplex structure were unusual [23].

### K^+^ facilitates the G-quadruplex formation in the g3-5 sequence

**Figure 4:**
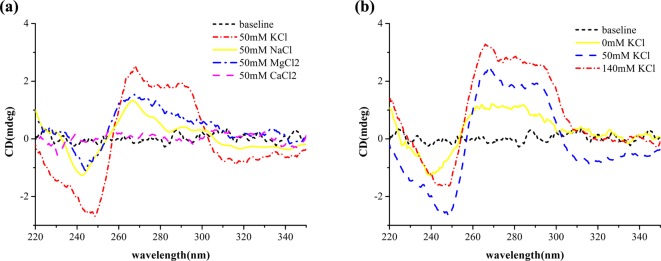
CD spectra of g3-5 in the presence of different cations (**a**) CD spectra of g3-5 in the presence of 50 mM KCl, NaCl, MgCl_2_, and CaCl_2_. (**b**) CD spectra of g3-5 in the different concentration of KCl (0–140 mM).

Cations played an important role in changing the G-quadruplex topology structure and its stability. The sequence of AG_3_TTAG_3_TTAG_3_TTAG_3_ adopted a basket-type antiparallel G-quadruplex structure in Na^+^ solution [24,25]. However, it folded into a hybrid antiparallel/parallel G-quadruplex structure in K^+^ solution [25–27]. In addition, the native thrombin binding aptamer (TBA) in Li^+^, Na^+^, Cs^+^, Ca^2+^, and Mg^2+^ solutions showed the same CD spectra with the K^+^ condition [28]. No matter what topological structure TBA forms, K^+^, Na^+^, Mg^2+^, and Ca^2+^ can stabilize the G-quadruplex structure [29–33]. To explore the effects of metal ions on the g3-5 G-quadruplex formation, their CD spectra were recorded in KCl, NaCl, MgCl_2_, or CaCl_2_ solution. A similar CD spectra of g3-5 with lower peaks, were found in the Na^+^ and Mg^2+^ buffer, as well as the K^+^ buffer (Figure 4a). However, the G-quadruplex structure of g3-5 disappeared in the Ca^2+^ buffer (Figure 4a). The CD spectrum of g3-5 in 140 mM KCl solution was stronger than that in 50 mM KCl solution (Figure 4b), implying that 140 mM K^+^ promoted formation of G-quadruplex structure. In contrast, CD spectra of g3-5 in 200 mM Mg^2+^ and Na^+^ showed weaker profiles (Supplementary Figure S2) than that in 50 mM buffer. However, the melting and annealing processes of g3-5 at 295 nm in the buffer of Mg^2+^ or Na^+^ exhibited no regular characteristic hypochromic curves (data not shown), which meant no stable G-quadruplex structure. Taken together, the strong elliptic signal of g3-5 CD spectrum in K^+^ solution supported that K^+^ favored the formation of the hybrid antiparallel/parallel G-quadruplex structure [34].

**Figure 5:**
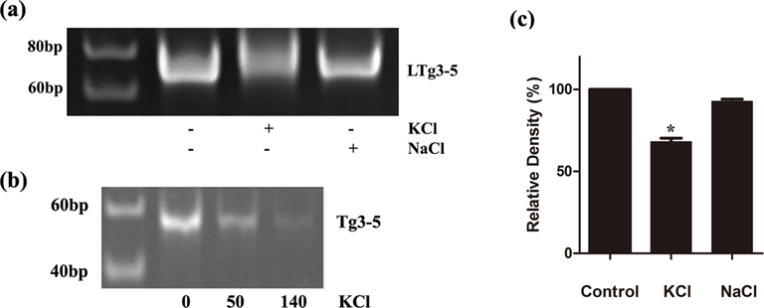
Klenow fragment polymerase stop assay of LTg3-5 and Tg3-5 (**a**) Polymerase stop assay results of LTg3-5 in the presence of 50 mM K^+^ and 50 mM Na^+^ respectively. (**b**) Polymerase stop assay results of Tg3-5 in the presence of 50 and 140 mM K^+^. The lane 1 represents the size marker. (**c**) Quantitation of the products of template LTg3-5 in Figure (a). Significant differences are indicated by an asterisk.

G-quadruplex structure can arrest DNA extension [14,31,35–37]. DNA polymerase stop assay was performed to further test the key role of K^+^ on the G-quadruplex formation and the effect of G-quadruplex structure on DNA extension. We used the long tail template LTg3-5 and the short one Tg3-5, after considering spatial hindrance of the additional tail in the formation of G-quadruplex structure (Table 2). We found both of them could form partial hybrid double-strands with the primer. The hybrid double-strand was then bidirectionally extended by Klenow fragment at room temperature [20]. The extension products were analyzed with 15% polyacrylamide gel. There was no difference between these two templates on the formation of G-quadruplex structure. The extension products of LTg3-5 in the NaCl buffer were similar to those in the control buffer (Figure 5a,c). However, KCl significantly reduced the products of LTg3-5 (Figure 5a,c). It was revealed that K^+^ could facilitate the G-quadruplex formation of g3-5 to stop DNA extension, but Na^+^ could not. Moreover, extension products were decreasing with the increasing concentration of K^+^ (Figure 5b). The results were entirely consistent with our CD and UV spectral data. In our previous study, CD spectra and polymerase stop assay results revealed that K^+^ was essential for the formation of Pu23WT G-quadruplex structure in *MET* promoter and the inhibitory effect of DNA polymerization [20]. Therefore, it may be assumed that there existed two mixed types of G-quadruplex structures folded in equilibrium by g3-1/Pu23WT and g3-5 in cells where the concentration of K^+^ was high enough.

### TMPyP4 attenuates the polymerization products by stabilizing the g3-5 G-quadruplex structure

**Figure 6:**
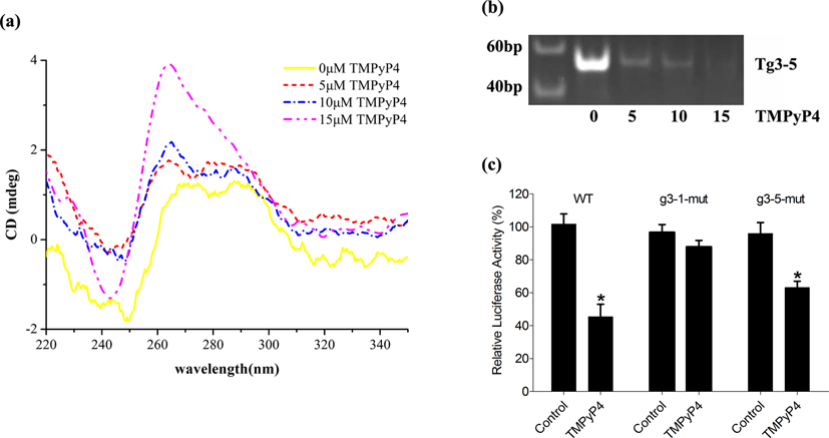
Inhibitory effect of g3-5 G-quadruplex (**a**) CD spectra of g3-5 in the TMPyP4-containing buffer at different concentrations. (**b**) Polymerase stop assay of Tg3-5 in the presence of TMPyP4. The lane 1 shows the molecular-weight size marker. (**c**) Relative firefly luciferase activities of the 0.7met-pGL4.10 plasmid and its site-directed mutations in HepG2 cells. Significant differences are indicated by an asterisk.

It has been demonstrated that the cationic porphyrin TMPyP4 can stabilize the G-quadruplex structure [38–41]. In order to assess the effect of TMPyP4, CD spectra, polymerase stop assay, and *MET*-dependent firefly luciferase activities of g3-5 strand were analyzed. CD spectra of g3-5 hybrid antiparallel/parallel G-quadruplex structure became more dominant when TMPyP4 was added into the buffer (Figure 6a). The stronger elliptic peak arose in the higher concentration of TMPyP4, which indicated a TMPyP4 concentration-dependent G-quadruplex stability of g3-5. Furthermore, the effect of TMPyP4 on Tg3-5 extension was analyzed using DNA polymerase stop assay (Figure 6b). It showed that many extension products of Tg3-5 existed in the absence of TMPyP4. However, the extension products of Tg3-5 were decreasing with the increasing concentration of TMPyP4. The products almost disappeared in the 15 μM TMPyP4 (Figure 6b). We also assessed the G-quadruplex function of g3-5 in cells. 0.7met-pGL4.10 is the *MET*-dependent firefly luciferase recombinant plasmid, whose insertion fragment is the promoter sequence of the *MET* gene located at −662 to +47 [20], where it was named WT. The mutants of WT were then constructed according to Table 3. The transfected HepG2 cells were treated with 50 μM TMPyP4 for 24 h, after which the relative firefly luciferase activities were detected. The relative luciferase activities of WT plasmid in the transfected HepG2 cells decreased significantly to 44.72% due to the stable G-quadruplex structure (Figure 6c). However, TMPyP4 had little effect on the g3-1-mut plasmid. But TMPyP4 still reduced the relative firefly luciferase activity to approximately 65.84% in the g3-5-mut plasmid. Therefore, it was concluded that the full *MET* promoter retained the G-quadruplex structure, and g3-1/Pu23WT G-quadruplex was the primary regulatory element for *MET* proto-oncogene expression in the cells. Though the G-quadruplex structure of g3-5 arrested DNA extension *in vitro*, g3-5 strands in the g3-1-mut plasmid could not respond to the treatment of TMPyP4. It is possible there could exist an interplay between g3-1/Pu23WT and g3-5 G-quadruplex structures due to a steric effect in recombinant plasmid. The *T*_m_ value of g3-5 was 47.46 ± 0.27°C in 140 mM KCl, which was much lower than that of g3-1/Pu23WT (81.07 ± 0.35°C) [20]. Hence, the g3-1/Pu23WT G-quadruplex structure was much more stable than g3-5 G-quadruplex. Furthermore, there was one Sp1 binding site located in g3-1/Pu23WT, which bound to Sp1 factor to initiate *MET* transcription [42,43]. The stable G-quadruplex of g3-1/Pu23WT strongly prevented the binding of Sp1 factor to its binding site, resulting in a decrease in gene transcription. Therefore, the G-quadruplex structure in g3-1/Pu23WT fragment played a crucial role in *MET* expression.

Our study revealed that the g3-5 strand adopted an intramolecular hybrid antiparallel/parallel G-quadruplex structure. And the G-quadruplex could stop DNA polymerization reaction in K^+^ and TMPyP4-containing buffer *in vitro*, although its effect on gene expression in HepG2 cells was very weak.

## Conclusion

In the present study, we found a novel G-quadruplex motif formed in the Human *MET* promoter region. The G-rich strand g3-5 could fold into an intramolecular hybrid antiparallel/parallel G-quadruplex structure under physiological conditions *in vitro*. The G-quadruplex structure in g3-5 strongly arrested the DNA extension in the presence of K^+^ and TMPyP4. However, this G-quadruplex structure could not decrease the luciferase activities in HepG2 cells. All the results showed that there existed a stable G-quadruplex structure in g3-5 *in vitro*, which could not act as a repressive element when it coexisted with the more stable g3-1/Pu23WT G-quadruplex structure. The findings in the present study will enrich our understanding about the G-quadruplex formation and gene expression regulation in the proto-oncogene promoters.

## Supporting information

**Fig S1:** Melting-annealing profiles of the truncations at 295 nm.

**Fig S2:** CD spectra of g3-5 at the high concentration of KCl, NaCl, MgCl2 and CaCl2.

## Abbreviations

CD: circular dichroism
G: guanine
HGF: hepatocyte growth factor
HGFR: hepatocyte growth factor receptor
TBA: thrombin binding aptamer
TMPyP4: 5,10,15,20-Tetra-(N-methyl-4-pyridyl) porphine

## References

[1] Peruzzi B. and Bottaro D.P. (2006) Targeting the c-Met signaling pathway in cancer. Clin. Cancer Res. 12, 3657–3660 16778093 10.1158/1078-0432.CCR-06-0818

[2] Birchmeier C., Birchmeier W., Gherardi E. and Vande Woude G.F. (2003) Met, metastasis, motility and more. Nat. Rev. Mol. Cell Biol. 4, 915–925 14685170 10.1038/nrm1261

[3] You W.-K. and McDonald D.M. (2008) The hepatocyte growth factor/c-Met signaling pathway as a therapeutic target to inhibit angiogenesis. BMB Rep. 41, 833–839 19123972 10.5483/bmbrep.2008.41.12.833 PMC4417610

[4] Cipriani N.A., Abidoye O.O., Vokes E. and Salgia R. (2009) MET as a target for treatment of chest tumors. Lung Cancer 63, 169–179 18672314 10.1016/j.lungcan.2008.06.011 PMC2659375

[5] Bean J., Brennan C., Shih J.-Y., Riely G., Viale A., Wang L. (2007) MET amplification occurs with or without T790M mutations in EGFR mutant lung tumors with acquired resistance to gefitinib or erlotinib. Proc. Natl. Acad. Sci. U.S.A. 104, 20932–20937 18093943 10.1073/pnas.0710370104 PMC2409244

[6] Cañadas I., Rojo F., Arumí-Uría M., Rovira A., Albanell J. and Arriola E. (2010) C-MET as a new therapeutic target for the development of novel anticancer drugs. Clin. Transl. Oncol. 12, 253–260 20462834 10.1007/s12094-010-0501-0

[7] Christensen J.G., Burrows J. and Salgia R. (2005) c-Met as a target for human cancer and characterization of inhibitors for therapeutic intervention. Cancer Lett. 225, 1–26 15922853 10.1016/j.canlet.2004.09.044

[8] Burge S., Parkinson G.N., Hazel P., Todd A.K. and Neidle S. (2006) Quadruplex DNA: sequence, topology and structure. Nucleic Acids Res. 34, 5402–5415 17012276 10.1093/nar/gkl655 PMC1636468

[9] Patel D.J., Phan A.T. and Kuryavyi V. (2007) Human telomere, oncogenic promoter and 5′-UTR G-quadruplexes: diverse higher order DNA and RNA targets for cancer therapeutics. Nucleic Acids Res. 35, 7429–7455 17913750 10.1093/nar/gkm711 PMC2190718

[10] Todd A.K., Haider S.M., Parkinson G.N. and Neidle S. (2007) Sequence occurrence and structural uniqueness of a G-quadruplex in the human c-kit promoter. Nucleic Acids Res. 35, 5799–5808 17720713 10.1093/nar/gkm609 PMC2034477

[11] Siddiqui-Jain A., Grand C.L., Bearss D.J. and Hurley L.H. (2002) Direct evidence for a G-quadruplex in a promoter region and its targeting with a small molecule to repress c-MYC transcription. Proc. Natl. Acad. Sci. U.S.A. 99, 11593–11598 12195017 10.1073/pnas.182256799 PMC129314

[12] Sun D., Guo K., Rusche J.J. and Hurley L.H. (2005) Facilitation of a structural transition in the polypurine/polypyrimidine tract within the proximal promoter region of the human VEGF gene by the presence of potassium and G-quadruplex-interactive agents. Nucleic Acids Res. 33, 6070–6080 16239639 10.1093/nar/gki917 PMC1266068

[13] Dexheimer T.S., Sun D. and Hurley L.H. (2006) Deconvoluting the structural and drug-recognition complexity of the G-quadruplex-forming region upstream of the bcl-2 P1 promoter. J. Am. Chem. Soc. 128, 5404–5415 16620112 10.1021/ja0563861 PMC2580050

[14] De Armond R., Wood S., Sun D., Hurley L.H. and Ebbinghaus S.W. (2005) Evidence for the presence of a guanine quadruplex forming region within a polypurine tract of the hypoxia inducible factor 1α promoter. Biochemistry 44, 16341–16350 16331995 10.1021/bi051618u

[15] Guo K., Pourpak A., Beetz-Rogers K., Gokhale V., Sun D. and Hurley L.H. (2007) Formation of pseudosymmetrical G-quadruplex and i-motif structures in the proximal promoter region of the RET oncogene. J. Am. Chem. Soc. 129, 10220–10228 17672459 10.1021/ja072185g PMC2566970

[16] Cogoi S. and Xodo L.E. (2006) G-quadruplex formation within the promoter of the KRAS proto-oncogene and its effect on transcription. Nucleic Acids Res. 34, 2536–2549 16687659 10.1093/nar/gkl286 PMC1459413

[17] Qin Y., Rezler E.M., Gokhale V., Sun D. and Hurley L.H. (2007) Characterization of the G-quadruplexes in the duplex nuclease hypersensitive element of the PDGF-A promoter and modulation of PDGF-A promoter activity by TMPyP4. Nucleic Acids Res. 35, 7698–7713 17984069 10.1093/nar/gkm538 PMC2190695

[18] Mikami-Terao Y., Akiyama M., Yuza Y., Yanagisawa T., Yamada O. and Yamada H. (2008) Antitumor activity of G-quadruplex-interactive agent TMPyP4 in K562 leukemic cells. Cancer Lett. 261, 226–234 18096315 10.1016/j.canlet.2007.11.017

[19] Hurley L.H., Von Hoff D.D., Siddiqui-Jain A. and Yang D. (2006) Drug targeting of the c-MYC promoter to repress gene expression via a G-quadruplex silencer element. Semin. Oncol. 33, 498–512 16890804 10.1053/j.seminoncol.2006.04.012

[20] Yan J., Zhao X., Liu B., Yuan Y. and Guan Y. (2016) An intramolecular G-quadruplex structure formed in the human MET promoter region and its biological relevance. Mol. Carcinog. 55, 897–909 25945949 10.1002/mc.22330

[21] Mergny J.-L. and Lacroix L. (2003) Analysis of thermal melting curves. Oligonucleotides 13, 515–537 15025917 10.1089/154545703322860825

[22] Ou T.-M., Lu Y.-J., Zhang C., Huang Z.-S., Wang X.-D., Tan J.-H. (2007) Stabilization of G-quadruplex DNA and down-regulation of oncogene c-myc by quindoline derivatives. J. Med. Chem. 50, 1465–1474 17346034 10.1021/jm0610088

[23] Qin Y. and Hurley L.H. (2008) Structures, folding patterns, and functions of intramolecular DNA G-quadruplexes found in eukaryotic promoter regions. Biochimie 90, 1149–1171 18355457 10.1016/j.biochi.2008.02.020 PMC2585383

[24] Wang Y. and Patel D.J. (1993) Solution structure of the human telomeric repeat d[AG3(T2AG3)3] G-tetraplex. Structure 1, 263–282 8081740 10.1016/0969-2126(93)90015-9

[25] Xu Y., Noguchi Y. and Sugiyama H. (2006) The new models of the human telomere d[AGGG(TTAGGG)3] in K+ solution. Bioorg. Med. Chem. 14, 5584–5591 16682210 10.1016/j.bmc.2006.04.033

[26] Ambrus A., Chen D., Dai J., Bialis T., Jones R.A. and Yang D. (2006) Human telomeric sequence forms a hybrid-type intramolecular G-quadruplex structure with mixed parallel/antiparallel strands in potassium solution. Nucleic Acids Res. 34, 2723–2735 16714449 10.1093/nar/gkl348 PMC1464114

[27] Dai J., Dexheimer T.S., Chen D., Carver M., Ambrus A., Jones R.A. (2006) An intramolecular G-quadruplex structure with mixed parallel/antiparallel G-strands formed in the human BCL-2 promoter region in solution. J. Am. Chem. Soc. 128, 1096–1098 16433524 10.1021/ja055636a PMC2556172

[28] Kankia B.I. and Marky L.A. (2001) Folding of the thrombin aptamer into a G-quadruplex with Sr(2+): stability, heat, and hydration. J. Am. Chem. Soc. 123, 10799–10804 11686680 10.1021/ja010008o

[29] Huang W., Smaldino P.J., Zhang Q., Miller L.D., Cao P., Stadelman K. (2012) Yin Yang 1 contains G-quadruplex structures in its promoter and 5′-UTR and its expression is modulated by G4 resolvase 1. Nucleic Acids Res. 40, 1033–1049 21993297 10.1093/nar/gkr849 PMC3273823

[30] Gros J., Rosu F., Amrane S., De Cian A., Gabelica V., Lacroix L. (2007) Guanines are a quartet’s best friend: impact of base substitutions on the kinetics and stability of tetramolecular quadruplexes. Nucleic Acids Res. 35, 3064–3075 17452368 10.1093/nar/gkm111 PMC1888817

[31] Yan Y.Y., Lin J., Ou T.M., Tan J.H., Li D., Gu L.Q. (2010) Selective recognition of oncogene promoter G-quadruplexes by Mg2+. Biochem. Biophys. Res. Commun. 402, 614–618 20971074 10.1016/j.bbrc.2010.10.065

[32] Miyoshi D., Nakao A. and Sugimoto N. (2003) Structural transition from antiparallel to parallel G-quadruplex of d(G4T4G4) induced by Ca2+. Nucleic Acids Res. 31, 1156–1163 12582234 10.1093/nar/gkg211 PMC150229

[33] Zhao X., Liu B., Yan J., Yuan Y., An L. and Guan Y. (2014) Structure variations of TBA G-quadruplex induced by 2′-O-methyl nucleotide in K+ and Ca2+ environments. Acta Biochimica et Biophysica Sinica 46, 837–850 25246433 10.1093/abbs/gmu077

[34] Guo K., Gokhale V., Hurley L.H. and Sun D. (2008) Intramolecularly folded G-quadruplex and i-motif structures in the proximal promoter of the vascular endothelial growth factor gene. Nucleic Acids Res. 36, 4598–4608 18614607 10.1093/nar/gkn380 PMC2504309

[35] Weitzmann M.N., Woodford K.J. and Usdin K. (1996) The development and use of a DNA polymerase arrest assay for the evaluation of parameters affecting intrastrand tetraplex formation. J. Biol. Chem. 271, 20958–20964 8702855 10.1074/jbc.271.34.20958

[36] Han H., Hurley L.H. and Salazar M. (1999) A DNA polymerase stop assay for G-quadruplex-interactive compounds. Nucleic Acids Res. 27, 537–542 9862977 10.1093/nar/27.2.537 PMC148212

[37] Qin Y., Rezler E.M., Gokhale V., Sun D. and Hurley L.H. (2007) Characterization of the G-quadruplexes in the duplex nuclease hypersensitive element of the PDGF-A promoter and modulation of PDGF-A promoter activity by TMPyP4. Nucleic Acids Res. 35, 7698–7713 17984069 10.1093/nar/gkm538 PMC2190695

[38] Han H., Langley D.R., Rangan A. and Hurley L.H. (2001) Selective interactions of cationic porphyrins with G-quadruplex structures. J. Am. Chem. Soc. 123, 8902–8913 11552797 10.1021/ja002179j

[39] Sun D., Guo K. and Shin Y.-J. (2011) Evidence of the formation of G-quadruplex structures in the promoter region of the human vascular endothelial growth factor gene. Nucleic Acids Res. 39, 1256–1265 20959293 10.1093/nar/gkq926 PMC3045601

[40] Arora A. and Maiti S. (2009) Differential biophysical behavior of human telomeric RNA and DNA quadruplex. J. Phys. Chem. B 113, 10515–10520 19572668 10.1021/jp810638n

[41] Parkinson G.N., Ghosh R. and Neidle S. (2007) Structural basis for binding of porphyrin to human telomeres. Biochemistry 46, 2390–2397 17274602 10.1021/bi062244n

[42] Zhang X., Li Y., Dai C., Yang J., Mundel P. and Liu Y. (2003) Sp1 and Sp3 transcription factors synergistically regulate HGF receptor gene expression in kidney. Am. J. Physiol. Renal. Physiol. 284, F82–94 12473536 10.1152/ajprenal.00200.2002

[43] Liang H., O’Reilly S., Liu Y., Abounader R., Laterra J., Maher V.M. (2004) Sp1 regulates expression of MET, and ribozyme-induced down-regulation of MET in fibrosarcoma-derived human cells reduces or eliminates their tumorigenicity. Int. J. Oncol. 24, 1057–1067 15067326

