# Supplementary material for: A novel G-quadruplex motif in the Human MET promoter region
Source: Biosci Rep. 2017 Nov 29;37(6):BSR20171128. doi: 10.1042/BSR20171128 (PMC5705779; doi:10.1042/BSR20171128)
Supplement: Supplementary file 1 [file bsr20171128_Supp1.pdf]

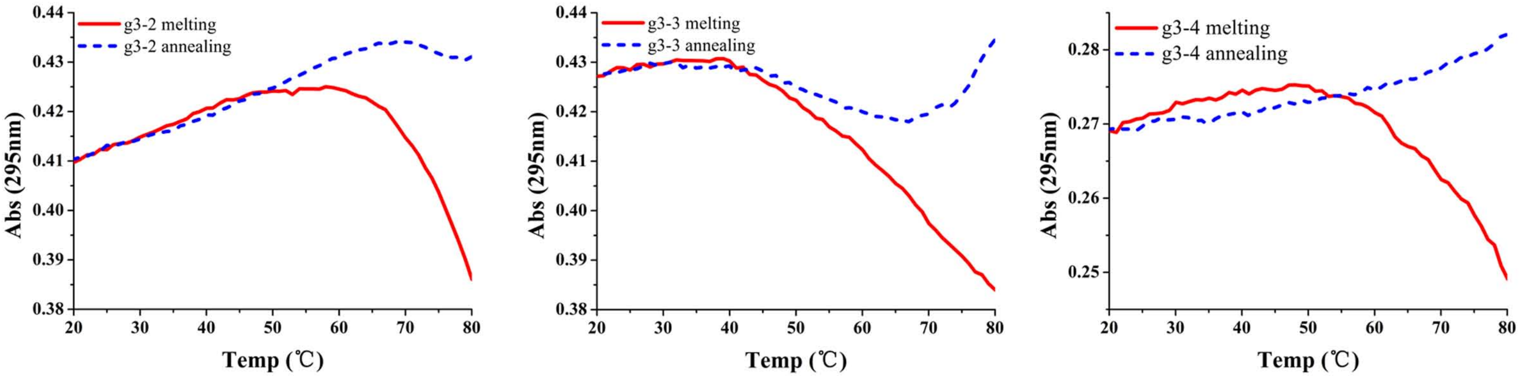

Fig. S1. Melting-annealing profiles of the truncations at 295 nm

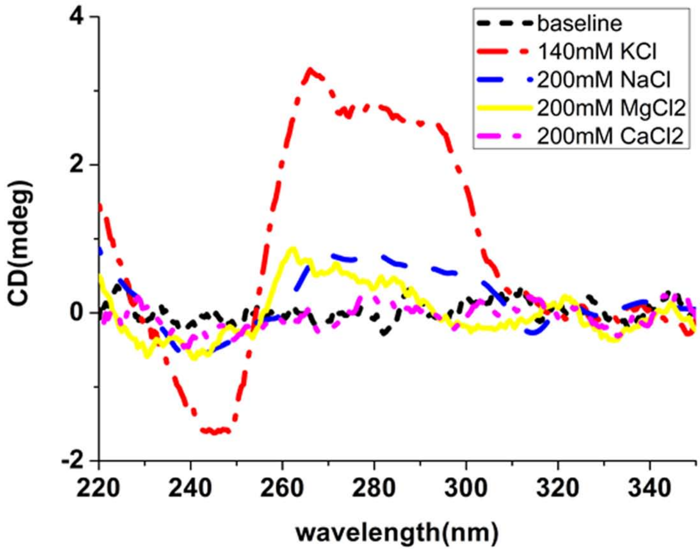

Fig. S2. CD spectra of g3-5 at the high concentration of KCl, NaCl, MgCl2 and CaCl2
